# Hospital nurse understaffing and short work experience: associations with mortality among patients

**DOI:** 10.1093/eurpub/ckac129.179

**Published:** 2022-10-25

**Authors:** L Peutere, J Pentti, A Ropponen, M Kivimäki, M Härmä, O Krutova, J Ervasti, A Koskinen, M Virtanen

**Affiliations:** 1 School of Educational Sciences and Psychology, University of Eastern Finland, Joensuu, Finland; 2 Clinicum, Department of Public Health, University of Helsinki, Helsinki, Finland; 3 Department of Public Health, University of Turku, Turku, Finland; 4 Research and Service Unit, Finnish Institute of Occupational Health, Helsinki, Finland; 5 Division of Insurance Medicine, Karolinska Institutet, Stockholm, Sweden; 6 Department of Epidemiology & Public Health, University College London, London, UK

## Abstract

**Background:**

Determining and maintaining optimal staffing level in hospitals is crucial, as understaffing may have serious consequences and even increase mortality risk among patients. There is no consensus, however, on the optimal way to determine staffing requirements in hospitals as patients’ care needs vary between wards and days. Nurse work experience may also affect quality of care and ultimately patients’ survival but research on this topic is scarce.

**Methods:**

Administrative register data on patients (N = 254,308) and employees of 40 hospital units was used in one hospital district in Finland from years 2013-2019. Both nurse understaffing and nurse work experience were measured with two different indicators in each unit-day. Mixed-effects survival models were used to analyse the associations of these exposures with mortality at patient-level, when adjusted for patients’ characteristics, such as age, sex and comorbidities.

**Results:**

Preliminary results showed that every one percent increase in the cumulative proportion of understaffed days - measured as low nursing hours relative to planned - was associated with 1.002-fold mortality risk among patients (95% CI, 1.000-1.004, p-value=0.044). Short work experience was not associated with increased risk of death.

**Conclusions:**

This study supports previous findings on the associations between nurse understaffing and increased mortality risk among patients in Finland although no association with mortality was found for the other three staffing characteristics. However, the average daily shares of actualized nursing hours relative to planned hours were quite high in hospital units. An indicator based on actualized relative to planned working hours in routine administrative data could be useful in evaluating understaffing in hospitals.

**Key messages:**

• Adequate level of nursing professional in hospitals is related to patient survival.

• It is also crucial of develop efficient ways to evaluate understaffing in hospitals.

